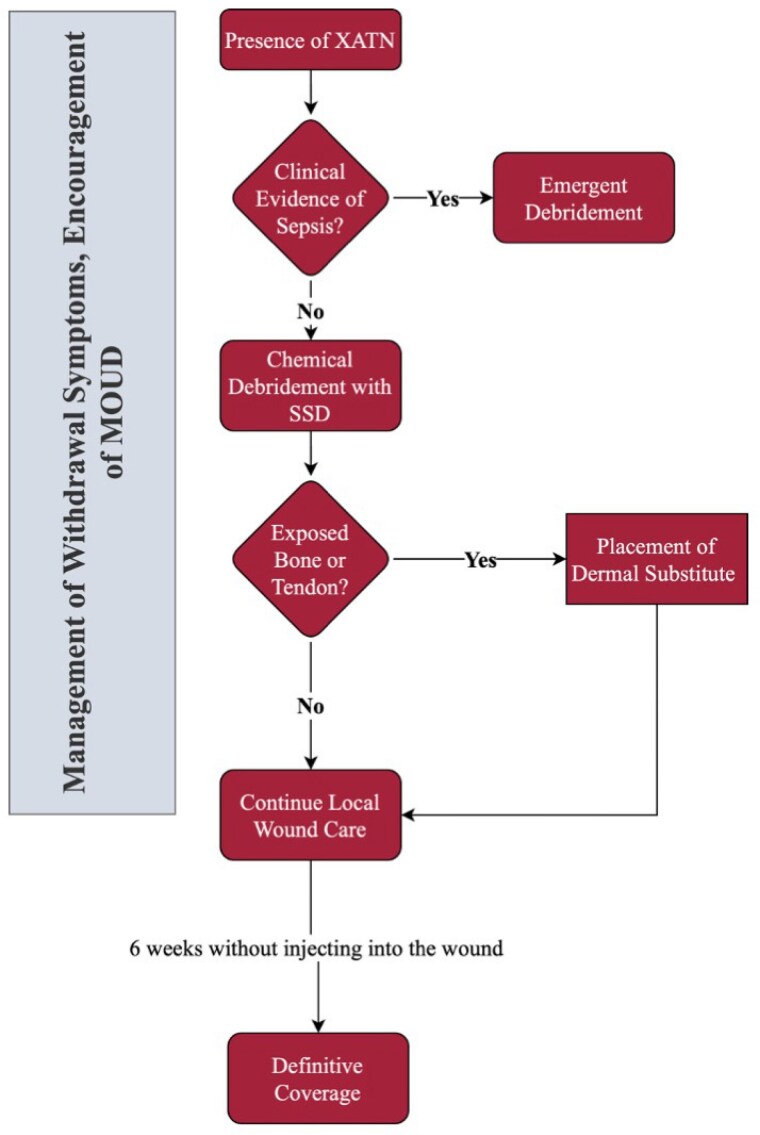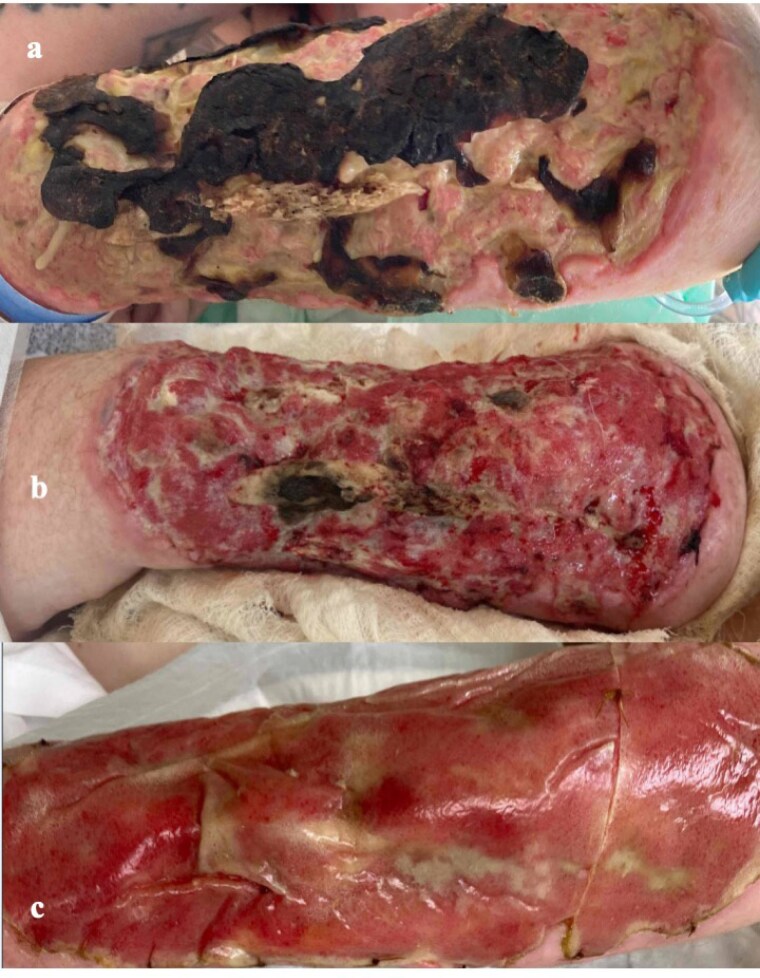# 524 Application of Burn Surgical Principles to the Management of Xylazine Associated Tissue Necrosis

**DOI:** 10.1093/jbcr/iraf019.153

**Published:** 2025-04-01

**Authors:** Aron Egelko, Miranda Haslam, Nathan Doremus, Jacob Menzer, Lisa Rae

**Affiliations:** Temple University Hospital; Temple University Hospital; Temple University Hospital; Temple University Hospital; Temple University Hospital

## Abstract

**Introduction:**

Street fentanyl is frequently adulterated with xylazine. A high prevalence of necrotic wounds (Xylazine Associated Tissue Necrosis; XATN) has been found in people who inject drugs. We have seen an increased prevalence of these wounds since 2019. XATN involves dead tissue with infection and burn centers are well versed in treatment of necrotizing soft tissue infections, burn wound infections, and large soft tissue deficits with exposed bone and tendon. Starting in August 2023, our regional burn center formalized a workflow for patients at high risk of limb loss which applied burn surgical principles to manage these wounds (Figure 1).

**Methods:**

This is a single-center retrospective chart review of patients with XATN pre-pathway from 1/2021- 8/2023 and post-pathway from 8/2023 - 6/2024. Starting in 8/2023, patients first underwent autolytic debridement with silver sulfadiazine for 5-7 days prior to operative treatment. Criteria for initial operative management included exposed bone and/or tendon. Patients underwent minimal debridement and placement of a dermal substitute to maintain tissue and to avoid or delay amputation

**Results:**

Pre-pathway, there were 338 admissions involving 86 patients. Patients were febrile on presentation for 17/338 (5%) admissions and hypotensive for 8/338 (2.4%). The median white blood cell count was 8.1 k/ml3 (IQR 6.4-11). Operative intervention was performed on 18/86 patients (20.9%), including 8 amputations. There were 6 deaths (7% of patients) none of which were attributable to the XATN. Post-pathway, 26 patients constituting 137 admissions were treated algorithmically. All had exposed tendon or bone and underwent successful dermal substitute placement (Figure 2), with a median follow-up of 152 days (IQR 91-255 days). On pre-operative admissions, median WBC count was 8.9 (IQR 7.7-11.4) and only 2/137 (1.5%) were febrile. Within the follow-up period, no patients presented with sepsis, only 1 patient (3.9%) underwent extremity amputation (due to a highly comminuted fracture within the XATN), 2 patients (7.7%) underwent delayed skin-grafting, and no patients died.

**Conclusions:**

Burn surgical principles can be safely and algorithmically applied to the management of XATN to achieve limb salvage without risk of septic progression.

**Applicability of Research to Practice:**

Initial radical debridement or amputation should be avoided for XATN in favor tissue preservation, autolytic debridement, and limb salvage techniques.

**Funding for the Study:**

N/A